# Recent advances in different interactions between toll-like receptors and hepatitis B infection: a review

**DOI:** 10.3389/fimmu.2024.1363996

**Published:** 2024-03-13

**Authors:** Saeed Soleiman-Meigooni, Aref Yarahmadi, Amir-Hossein Kheirkhah, Hamed Afkhami

**Affiliations:** ^1^ Infectious Diseases Research Center, Aja University of Medical Sciences, Tehran, Iran; ^2^ Department of Biology, Khorramabad Branch, Islamic Azad University, Khorramabad, Iran; ^3^ Department of Tissue Engineering and Applied Cell Sciences, School of Medicine, Qom University of Medical Sciences, Qom, Iran; ^4^ Nervous System Stem Cells Research Center, Semnan University of Medical Sciences, Semnan, Iran; ^5^ Cellular and Molecular Research Center, Qom University of Medical Sciences, Qom, Iran; ^6^ Department of Medical Microbiology, Faculty of Medicine, Shahed University, Tehran, Iran

**Keywords:** toll-like receptors (TLR), hepatitis B virus (HBV), polymorphism, immune system, signaling

## Abstract

Hepatitis B virus (HBV) B infections remain a primary global health concern. The immunopathology of the infection, specifically the interactions between HBV and the host immune system, remains somewhat unknown. It has been discovered that innate immune reactions are vital in eliminating HBV. Toll-like receptors (TLRs) are an essential category of proteins that detect pathogen-associated molecular patterns (PAMPs). They begin pathways of intracellular signals to stimulate pro-inflammatory and anti-inflammatory cytokines, thus forming adaptive immune reactions. HBV TLRs include TLR2, TLR3, TLR4, TLR7 and TLR9. Each TLR has its particular molecule to recognize; various TLRs impact HBV and play distinct roles in the pathogenesis of the disease. TLR gene polymorphisms may have an advantageous or disadvantageous efficacy on HBV infection, and some single nucleotide polymorphisms (SNPs) can influence the progression or prognosis of infection. Additionally, it has been discovered that similar SNPs in TLR genes might have varied effects on distinct populations due to stress, diet, and external physical variables. In addition, activation of TLR-interceded signaling pathways could suppress HBV replication and increase HBV-particular T-cell and B-cell reactions. By identifying these associated polymorphisms, we can efficiently advance the immune efficacy of vaccines. Additionally, this will enhance our capability to forecast the danger of HBV infection or the threat of dependent liver disease development via several TLR SNPs, thus playing a role in the inhibition, monitoring, and even treatment guidance for HBV infection. This review will show TLR polymorphisms, their influence on TLR signaling, and their associations with HBV diseases.

## 1 Introduction

Hepatitis B virus (HBV) infection is a universal public health concern and one of the prominent causes of severe and chronic liver damage worldwide (1). HBV is a member of the *Hepadnaviridae* family, and its virion includes a compact, semi-double-stranded, about 3.2 kb relaxed circular DNA (rcDNA) genome (2). HBV particularly infects hepatocytes and leads to acute liver diseases. The HBV replication is exclusive in that the genomic DNA is transformed to a molecular pattern DNA (covalently closed circular DNA: cccDNA) to augment a viral RNA mediator, which is next reverse-transcribed back to viral DNA. The extremely constant attributes of cccDNA lead to chronic infection and a weak level of treatment (3). Firstly, the viral polymerase gene has the largest open reading frame (ORF), and its protein has reverse transcription ability. Additionally, three viral envelope protein genes, comprising large (L), medium (M), and small (S) surface antigens, have been identified (HBsAg). In addition, the pre-core (also known as HBeAg) and core protein genes function as capsomers for the viral capsid. X (HBx) is a gene-encoded protein with regulatory roles. The most prevalent kind of liver cancer, hepatocellular carcinoma (HCC), is caused and developed in large part by the multifunctional regulatory protein known as HBx (4–6). HBV damages the liver through an immune response to infected liver cells. Immunosuppression increases replication and cytotoxicity. The severity of damage depends on virus-host interaction, leading to cirrhosis if not managed properly (7). HBV infection is primarily contracted through vertical transmission from mother to infant or horizontal transmission within the community during early childhood. In contrast, older children are at risk of HBV infection through exposure to contaminated blood during intravenous drug use or through sexual contact (8). HBV infection is the main factor of liver cirrhosis and HCC globally, with a yearly death of 1 million. About fifty percent of patients with chronic hepatitis B (CHB) infection will develop into liver cirrhosis, and the annual incidence of HCC in this group is four percent. The host immune reaction is the most crucial factor in HBV-related liver injury (4, 5, 9). Toll-like receptor (TLR) ligands can suppress HBV replication and transcription. TLR4 ligand LPS and TLR9 ligand cpG DNA are most effective against HBV replication. Meanwhile, TLR9 cpG DNA and TLR3 ligand dsRNA are effective against HBV RNA transcription. Reduced HBsAg expression was more prominent than the decrease of HBeAg. The group treated with TLR7 and TLR9 ligand cpG DNA showed the most pronounced suppression of released HBV antigen. The expression of HBc protein can be decreased by TLR4 ligand LPS, TLR9 ligand cpG DNA, and TLR3 ligand dsRNA (10). The purpose of this review is to show TLR response to HBV infection in human and animals, TLR polymorphisms, their influence on TLR signaling, and their associations with HBV diseases.

### 1.2 TLRs in innate immunity and pathogen recognition

**Figure 1:**
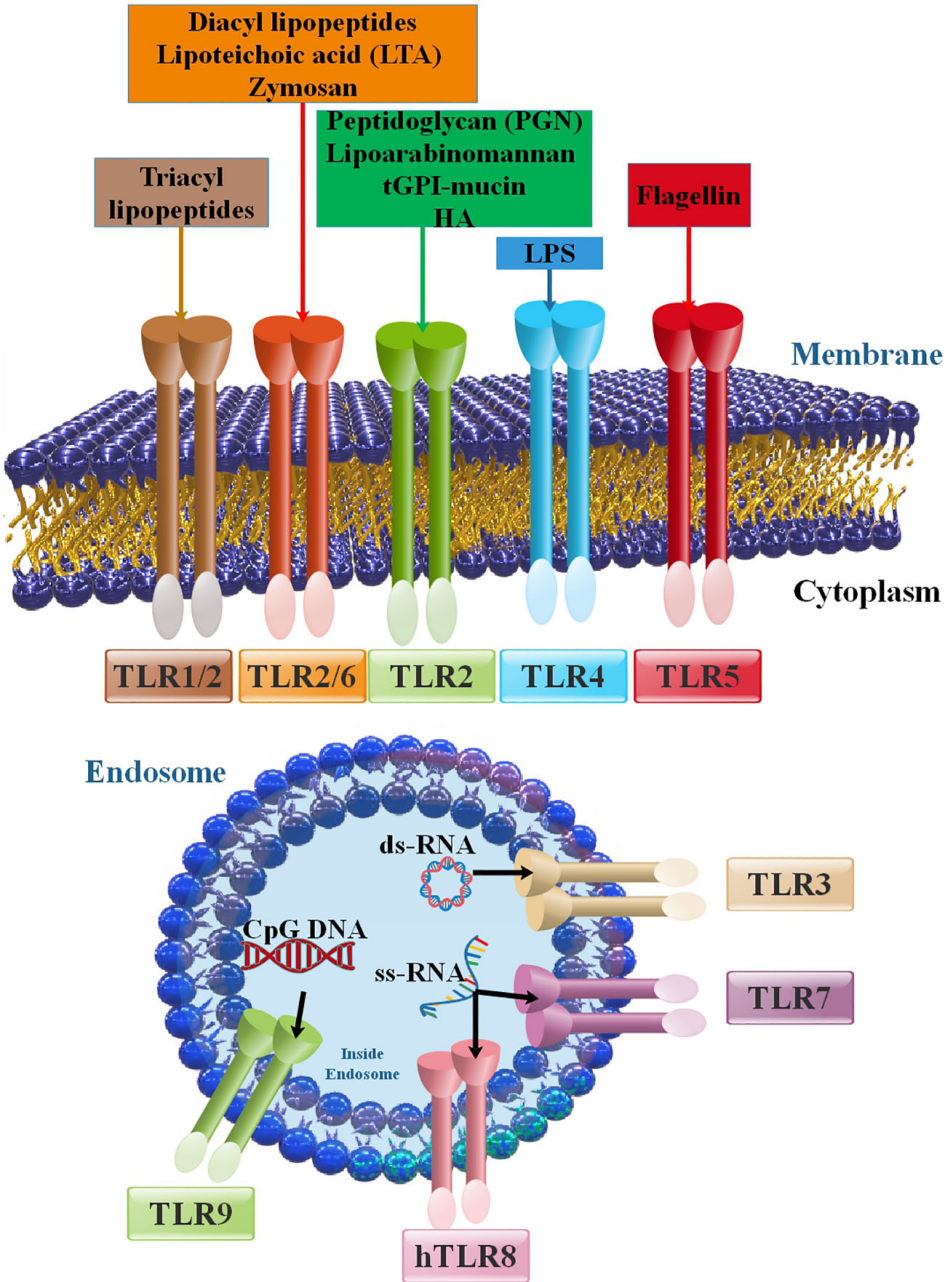
Members of the TLR family and their positions in cells, PAMPs, and DAMPs as inducers of TLRs. The principal attributes that recognize various TLRs are ligand property, signal transduction pathways, and subcellular localization. TLR ligands can be categorized into external and internal ligands according to their source. Cell membrane TLRs (including TLR2 with TLR1 and TLR6 in addition to TLR4, TLR5, and TLR10) that are expressed on the cell membrane. Intracellular TLRs or nucleic acids sensors (TLR3, TLR7, TLR8, and TLR9) are placed in the endoplasmic reticulum (ER), endosomes, and lysosomes (15, 16).

**Figure 2:**
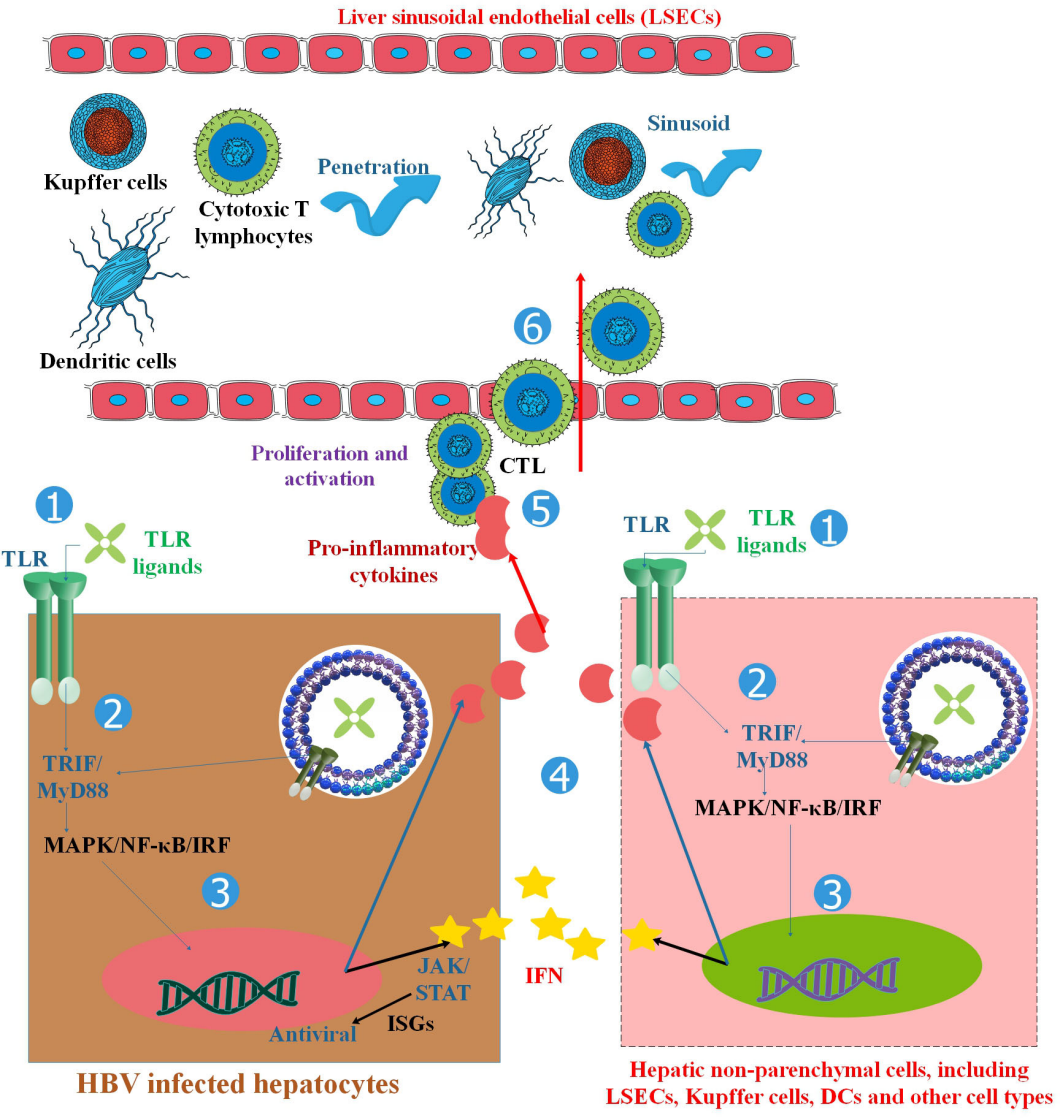
TLRs and the activation of antiviral innate and adaptive immune responses in HBV infection. TLRs are found in hepatocytes and non-parenchymal hepatic cells such as LSECs, Kupffer cells, Dendritic cells (DCs), and other cell types. TLR ligand stimulation activates downstream MyD88/TRIF-dependent signaling pathways in hepatic cells, resulting in the production of pro-inflammatory cytokines, chemokines, and IFNs. HBV replication can be inhibited in two ways: (1) the intracellular MAPK- and NF-κB-dependent signaling pathways trigger antiviral mechanisms, and (2) IFNs and other unknown antiviral factors stimulate the expression of ISGs and other antiviral activities in hepatocytes. Chemokines and inflammatory cytokines attract specific T cells into the liver, promote T-cell proliferation, and enhance HBV-specific CD8+ T-cell antiviral functions. By activating both innate and adaptive responses, TLRs inhibit HBV in the liver. Myeloid DCs respond to a broad spectrum of TLR ligands, such as those for TLR1/2, TLR-3, TLR-4, TLR-7, and TLR-9, to generate antiviral cytokines, which in turn suppress HBV replication in HBV-Met cells. Therefore, TLR triggering in hepatic NPCs and extra-hepatic DCs could remarkably decrease HBV replication by releasing IFN I or other antiviral cytokines (23).

Toll-like receptors (TLRs) can recognize highly conserved pathogen-associated molecular patterns (PAMPs), which function as agonists or TLR agonists/ligands (TLRLs). Specifically, TLRs on the cell surface are mostly responsible for identifying different components of microbial membranes, such as proteins, lipids, and lipoproteins (11). For instance, TLR5 recognizes extracellular flagellin and activates the production of pro-inflammatory cytokines. On the other hand, the Nod-like receptor (NLR) Ipaf recognizes flagellin in the cytoplasm of macrophages and activates caspase-1 (12). Several studies have shown that certain elements derived from the host, such as fibrinogen, heat shock proteins, RNA, and DNA, can also act as TLR ligands (13, 14). TLRs are expressed on cells implicated in the innate immune reaction (myeloid and NK cells) and several cells of the adaptive immune response (regulatory and triggered T cells) and intercede innate immune reactions versus microbial pathogens and stimulate adaptive immune reactions (13, 14). In the cell membrane, TLRs 1, 2, 4, 5, 6, and 10 are expressed. Lysosomes and endosomes include TLRs 3, 7, 8, and 9. TLRs 11, 12, and 13 are more active in other mammals. For instance, in mice, TLR11 plays a role in recognizing certain bacterial components (**Figure 1**). TLRs can recognize PAMPs of bacteria, viruses, and parasites based on their various kinds (17). Dendritic cells (DCs) and macrophages produce TLRs that specifically detect nucleic acids inside endosomes. TLR3, TLR7/8, and TLR9 are among the TLRs in this subgroup that are in charge of detecting, respectively, double-stranded (ds)RNA, single-stranded (ss)RNA, and ssDNA (18). Pattern recognition receptors (PRRs) belong to several families that monitor the cellular microenvironment for signs of viral infection. Viruses contain structural PAMPs, which are perfect targets for PRRs like TLRs. These receptors set off an intracellular signaling cascade that results in the production of cytokines and type-1 interferon (IFN), which drive the innate antiviral immune response. This defensive mechanism acts quickly while controlling the adaptive immune system (19, 20). The inflammatory cytokines induced by TLRs are associated with several hepatic disorders, such as inflammation of the liver, hepatic fibrosis, cirrhosis, alcohol-related liver disease (ARLD), non-alcoholic liver disease (NAFLD), reoxygenation injury, and HCC (21, 22). TLRs diagnose preserved PAMPs, which serve as TLRLs. TLRs can form homodimers or heterodimers, activated by certain ligands. Following ligand binding to TLRs, TLRs dimerize and change their conformation to attract downstream adaptor proteins, including myeloid differentiation primary response protein 88 (MyD88), TIRAP/MyD88-adaptor-like (Mal), TIR domain-containing adaptor inducing IFN-β (TRIF)/TIR domain-containing adaptor molecule-1 (TICAM-1), SARM (sterile *α* and armadillo-motif-containing protein), and TRF-related adaptor molecule (TRAM) (**Figure 2**) (24–30). The type of TLRs involved in dimerization depends on the type of PAMPs (17). TLRs tag along with one another following ligand binding. They are dimerized based on their TLR type. Most TLRs undergo homodimerization, including 3, 4, 5, 7, 8, and 9. However, the remainder is heterodimerized, such as TLR1/2, TLR2/6, and occasionally TLR7 and TLR8 (15, 31, 32). TLR4 is a well-known example of homodimerization; it is triggered by lipopolysaccharide (LPS) that is extracted from the outer membrane of Gram-negative bacteria, such as *Escherichia coli*. On the other hand, TLR2 can form heterodimers with TLR1 or TLR6, depending on the ligand (33). After the dimerization of TLRs, the signal might go along two major routes. The first, known as MyD88-dependent, employs the MyD88 mediator and leads to the yield of inflammatory cytokines such as tumor necrosis factor (TNF), IL-6, and IL-1, and chemokines such as C-C motif ligand 4, CCL4 (25, 26). The other pathway, TRIF-mediated, uses the TRIF mediator and eventuates the expression of type I IFNs like IFN*α*/*β* (34). TLR3 is the lonely TLR that triggers the TRIF-dependent path (35). Moreover, TLRs are associated with cell reproduction, survival, apoptosis, angiogenesis, tissue remodeling, repair, and the evolvement of innate and adaptive immune responses. These proteins become active through hetero or homodimerization (11, 36, 37).

## 2 TLR response to HBV infection in human

The fate of microbial infections, including viruses, is largely determined by TLRs, the most significant family of PRRs. For a quick antiviral response and to stop infection, early innate viral identification by host surface TLRs is crucial. Even though viruses can activate TLRs before host cell infection, the antiviral immune response is far more complex, involving various cell types such as T cells, B cells, NK cells, monocytes, and neutrophils. These cells can either promote infection upon TLR engagement or produce proinflammatory cytokines and chemokines to aid in removing the virus. By generating antimicrobial molecules like TNF-α and IFN, PAMP/microbe-associated molecular patterns (MAMPs)-induced activation of TLR signaling reduces the replication and spread of invading pathogens/microbes (38, 39). When HBV infection occurs, the TLR response may alter HBV-specific T and B cell responses, ending the HBV infection (23, 40). SNPs of TLRs may be crucial in the development of HBV, while the exact processes are still unknown (41). Peripheral blood mononuclear cells (PBMCs) from individuals with CHB showed noticeably decreased expression of TLR transcripts, including TLR1, 2, 4, and 6. Additionally, upon activation with TLR4 and TLR2 agonists, the cells had a reduced cytokine response that coincided with the patients’ plasma HBsAg levels, pointing to a potential link between HBsAg and TLR signaling (42). Another study demonstrated that PBMCs from children with chronic HBV infection were stimulated with ligands for TLR2, TLR3, and TLR9. This resulted in an increase in the production of IL-6, CCL3, and CXCL10, indicating the activation of TLR-mediated inflammatory response (43). However, the PBMCs from children with CHB revealed a considerably lower IFN-α production than those from healthy children upon stimulation with ligands for TLR2, TLR3, and TLR4, suggesting a suppressed IFN response (43). When PBMCs from CHB patients were compared to those from healthy controls, they showed a substantial drop in the expression of TLR signaling molecules, such as TRAF3, IRAK4, and IRF7. This finding suggests that individuals with chronic HBV infection have a compromised immune response (44). PBMCs from patients with CHB showed lower expression of both TLR7 mRNA and protein when compared to healthy controls; however, patients with CHB showed lower expression of TLR9 mRNA but higher levels of TLR9 protein, which correlated with their serum HBV DNA, indicating a potential connection between TLR9 protein expression and HBV replication (45).

### 2.1 TLR response in different animal models

TLR response to HBV infection in many animal models has been well investigated (46, 47). In animal models, TLR2 activation has been shown to expedite HBV clearance and improve HBV-specific T-cell responses (48). Isogawa et al. (49) conducted research whereby they observed that a single intravenous injection of TLR3, 4, 5, 7, and 9 agonists reduced HBV replication, hence inducing IFN-α/β in the liver of HBV-transgenic mice and indicating the anti-HBV effect of these TLRs. TLR-agonist-mediated regulation of HBV replication has also been shown in mouse non-parenchymal liver cells (50). The woodchuck model of CHB’s transcriptome study also showed a limited intrahepatic type I IFN response, like in individuals with CHB (51). A suppressive tendency of TLR expression was seen in the hepatocytes of the woodchuck model of chronic hepatitis compared to that of healthy animals. This finding suggests that the innate immune response is compromised in chronic infection (52). Yet, a recent study using a woodchuck model of hepatitis revealed the function of TLR2 in the resolution of HBV infection (48). Other recent research has shown, using an HBV mouse model, how TLR signaling enhances HBV-specific CD8+ T-cell responses, which regulate the infection (47). In an HBV hydrodynamic mouse model, intraperitoneal inoculation of 5,6-dimethylxanthenone-4-acetic acid (DMXAA), a STING agonist, induced type I IFNs that decreased HBV DNA replication intermediates in the mouse livers, demonstrating the role of the innate immune response in suppressing HBV replication (53). Tree shrews (Tupaia belangeri) be a useful animal model for HBV infection as HBV induces human-like symptoms in them, such as hepatitis and chronic infection. Tree shrews’ whole genomes have shown that they are more closely linked to humans than to rodents (54). Numerous studies have indicated that tree shrews are vulnerable to HBV infection (39). In research, Kayesh et al. (39) examined the infectivity of HBV-F infection in tree shrews, both wild-type and mutant (HBV-F Wt/Mt), and they also described the innate immune response against HBV infection in this model. found that HBV-F Wt/Mt may cause an acute infection in adult tree shrews, as evidenced by elevated alanine aminotransferase (ALT) levels in the sera and the presence of HBV DNA in the liver tissues of both 7 and 14 dpi-infected tree shrews. Only HBV-F-Wt-infected tree shrew liver tissues showed induction of IFN-β and upstream TLR1, 3, 7, 8, and 9, indicating a more stealthy form of HBV-F-Wt infection. TLR response characterization revealed several distinct responses between Mt and HBV-F-Wt, including downregulating TLR8 in Tupaia liver tissue infected with HBV-F-Wt. These variations may thus be related to the increased pathogenicity and replication activity of HBV-F-Mt compared to Wt; however, more research employing the tree shrew model is needed (39). Overall, these results point to a significant role that innate immunity plays in inhibiting HBV replication; further research is needed to determine the precise mechanisms (47).

## 3 Impact of HBV on TLRs

The precise processes through which HBV evades or inhibits the immune system remain unknown. In addition, it has not yet been determined whether HBV evades or suppresses innate recognition or boosts innate immunity. Nevertheless, HBV protein levels, notably HBsAg and HBeAg, are related to HBV survival, which suggests that viral proteins dampen the host immune response (55–57). Though the T-cell disorder is maybe the most important immune alteration that contributes to viral durability, HBV interplay with the innate immune response is presumably crucial, as the absence of efficient innate immunity has functional outcomes that promote chronic infection (58). Furthermore, the natural immune system influences T-cell responses and other adaptive immunological processes crucial for HBV control because of its intrinsic capacity to fight viral infections (59, 60). Although primary research *in vitro* showed that HBV could transiently stimulate an innate response in the infected cell, these outcomes might have been prevented via the absence of optimized model methods. In addition, novel investigations via Cheng et al. and Mutz et al. utilizing recently generated effective *in vitro* HBV infection methods showed that *in vitro* HBV infection does not induce a cell-intrinsic, innate immune response. In conclusion, HBV’s stealth trait allows it to evade inherent diagnosis in both its host organ and cell (61–63). Extracellular signal-regulated kinase 1/2 (ERK1/2), nuclear factor kappa B (NF-κB), and IRF-3 are all activated by HBV, which prevents hepatic cells from responding to TLR-mediated antiviral responses (64). By lowering TLR expression and cellular signaling pathways, HBV can inhibit the actions of TLR2/4 and TLR3 (65, 66). Research has demonstrated that in PBMCs from patients with CHB, the expression of TLR signaling molecules, such as TRAF3, IRAK4, and IRF7, was much lower than in healthy controls (44). It has been shown that HBV inhibits TLR9 response by suppressing the MyD88-IRAK4 route in Plasmacytoid dendritic cells (pDCs) isolated from patients (67). All peripheral B cell subsets exposed to HBV exhibited decreased TLR9 expression and B cell activity mediated by TLR9 (68). Additionally, peripheral DC subsets of people with persistent HBV infection show lower expressions of TLR8, 4, and 9. Reportedly, HBV polymerase inhibits IFN production by interfering with the interface between IκB kinase-” and DEAD-box RNA helicase, preventing IRF activation (25, 69, 70) (**Table 1**).

**Table 1:** Impact of signaling pathways of TLRs on HBV, such as the antiviral efficacy of TLR signaling against HBV.

TLRs	Pathway	Effects/functions	Ref
TLR2	TLR2 signaling in hepatocytes activates MAPK and PI-3 K/Akt pathways, which rely on adaptor molecules like TAK1, IRAK1/4, and TRAF6 for antiviral effects.	TLR2 signaling suppresses HBV replication in the absence of IFN. Using the dHepaRG cell model, Luangsay et al. found significant antiviral activity HBV in the attendance of TLR1/2, TLR4, and RIG-I/MDA-5 ligands, as well as the production of both cytokines IL-6 and IL-10.	(71, 72)
TLR3	The activation of TLR3 significantly upregulates the expression of the IFN-receptor in hepatocytes of HBS-B6 mice, resulting in increasing phosphorylation of transducers and activators of the STAT1-IRF-1 signaling cascade.	TLR3 signaling against HBV infection can be hindered by various cells generating IFN I, which impedes viral replication. Non-parenchymal cells produce IFN-β, which inhibits HBV replication through a TLR3 ligand.	(50, 73–76)
TLR4	TLR4 signaling reduces HBV replication via MAPK and PI-3 K/Akt. TLR signaling plays an important role in hepatocarcinogenesis as demonstrated by a decrease in liver cancer occurrence, size, and quantity in TLR4 and MyD88 deficient mice.	Intestinal microbiota and TLR4 are significant factors in Hepatocellular carcinoma. They promote cancer cell proliferation and reduce HBV replication. Wu et al. showed that TLR3 or TLR4-activated Kupffer cells combined with TLR3-activated LSECs led to soluble-molecule-dependent reduction of HBV replication *in vitro*.	(21, 22, 50, 71, 77, 78)
TLR7	TLR7 signaling and IFN-α/β receptor are crucial for CD81 T-cell activation and HBV clearance. TLR7 detects intracellular viral single-strand RNA, activating pro-inflammatory transcription factors through MYD88-dependent pathway.	GS-9620, a TLR7 agonist, reduces serum WHV DNA, hepatic replicative intermediates, and WHV cccDNA in woodchucks. It also eliminates serum WHsAg and may decrease the incidence of HCC. Its antiviral effectiveness was confirmed in an HBV-infected chimpanzee model.	(79–81)
TLR8	TLR8 is detectable in monocytes and mDCs. It detects ssRNA by binding to its secondary structures. The MyD88 pathway mediates TLR7 and TLR8 signaling by activating various proteins, leading to a potent inflammatory response and interferon induction.	TLRs and a mediated inflammatory response are necessary to prevent intrauterine transmission of HBV. Lack of TLR-intermediate response causes intrauterine HBV infection. Latest findings suggest that up-regulated TLR7 and TLR8 expression is crucial in the immunological response to HBV infection.	(82, 83)
TLR9	HBV can hinder TLR9 function by inhibiting the MyD88-IRAK4 axis and preventing the production of type I interferon in plasmacytoid dendritic cells and B lymphocytes. This could contribute to chronic infections.	HBV infection reduces pDCs’ IFN-α production in response to TLR9. HBV inhibits TLR9 signaling and transcriptional activity in pDCs and B cells, resulting in decreased TLR9 mRNA and protein levels. TLR9 mRNA and protein levels are lower in PBMC from patients with HBV-associated chronic hepatitis and HCC.	(67)

## 4 TLR polymorphisms

TLRs can diagnose various ingredient derivatives, mostly from viruses and other pathogens. Identified TLR ligands are listed and categorized according to their lipid, protein, and nucleic acid constituents. The role of TLR activation in innate and adaptive immune responses is supported in part by TLR polymorphisms, which have been shown to increase susceptibility to several infectious illnesses in humans (84). Gene polymorphisms, called single nucleotide polymorphism (SNP) and referred to as single nucleotide alterations in certain DNA regions inside the homologous interval, may originate from point mutations (85, 86). SNPs in the genes that encode proteins linked to innate responses have garnered a lot of interest, and several investigations have found SNPs in these genes in various animal species. Genetic code redundancy causes some SNPs, also known as synonymous polymorphisms, to not change any of the protein’s amino acids. In other situations (non-synonymous SNPs), the polymorphism results in a modified amino acid, which might not impact the structure or function of proteins (87). Significant progress in understanding innate immune system functions and genome-wide association studies (GWAS) have shown complex interactions among TLRs, genetic polymorphisms, and environmental variables (88).

## 5 Relation between TLR polymorphism and severity or susceptibility to the HBV

**Figure 3:**
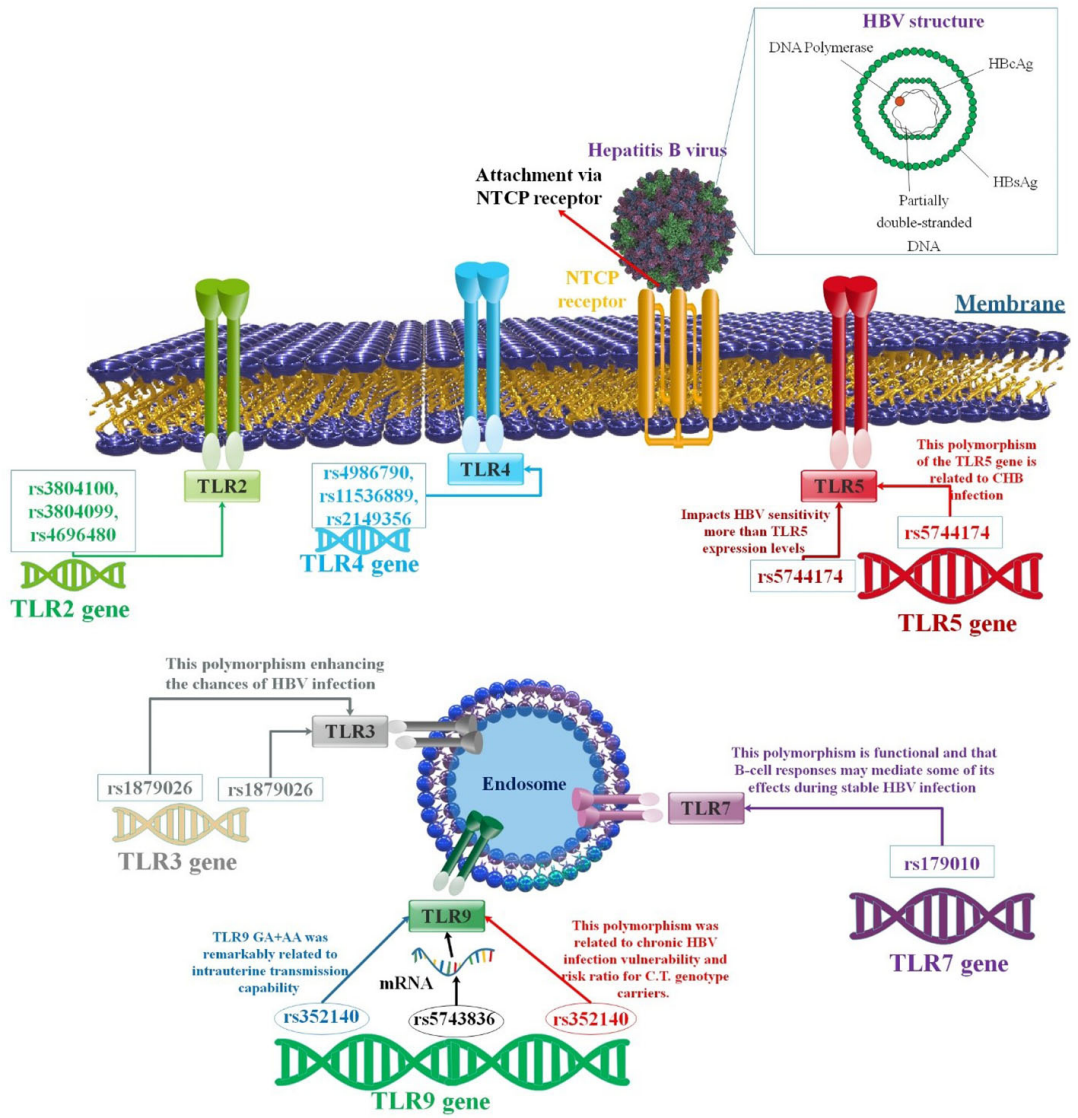
A correlation between several TLR polymorphisms and the progression of HBV infection. Variable SNPs may influence the passage or prevention of HBV infection. There have been significant investigations undertaken on the function of TLRs associated with immune reaction to HBV. Various TLRs have been related to suppressing HBV replication in human liver cells. Different findings showed that SNPs in the TLR genes could affect the outcome of HBV infection (94–96).

Numerous research studies show a correlation between TLR polymorphism and HBV infection (86, 89). The likelihood of contracting hepatitis B and its progression is influenced by the genetic characteristics of both the host and the virion and environmental, physiological, and metabolic factors. MicroRNAs (miRNAs) are becoming increasingly recognized as a significant determinant influencing several aspects of HBV infection. They regulate various biological processes, including inflammatory responses, cellular differentiation, proliferation, and programmed cell death. Additionally, extracellular miRNAs released by donor cells may be transferred into recipient cells by exosomes and extracellular vesicles, establishing a mechanism of communication between cells throughout a variety of standard and pathological activities (90). According to selective miRNA research conducted about TLR regulation, miRNAs may influence TLR signaling by acting as physiological ligands for TLRs or regulating transcription. Furthermore, research indicates that the TLR pathway can directly control miRNA expression (91). There exists a proposition suggesting that the presence of SNPs inside miRNA genes might potentially influence several aspects of miRNA functionality, including transcription, processing, and interactions with target mRNAs. Numerous research studies have been conducted to examine SNPs in miRNA genes to ascertain any potential links between HBV infection and the progression of HCC (92, 93). However, also, SNPs may positively or negatively impact HBV infection (41) (**Figure 3**). It is yet unclear how TLR polymorphism relates to the severity or vulnerability to HBV (93). On the other hand, some research has indicated that particular TLR variations could influence how an HBV infection turns out. For example, a study conducted in China found that TLR2 rs3804099 and TLR3 rs377529 polymorphisms were linked to a reduced risk of HBV-related HCC (97). TLR2 rs5743708 and TLR4 rs4986791 polymorphisms were linked to greater vulnerability to chronic HBV infection, but TLR9 rs187084 polymorphism was linked to lower susceptibility, according to another study (98, 99). In contrast, a study conducted in India found that TLR2 rs3804100 and TLR4 rs4986790 polymorphisms were associated with increased susceptibility to acute HBV infection, while TLR9 rs352139 polymorphism was associated with decreased susceptibility (99, 100).

### 5.1 TLR2

Chen et al. showed that serum anti-HBV response induced by vaccination was shown to be strongly related to four SNPs (rs2243248, rs1805015, rs1295686, and rs3804100) in the IL-4, IL-4RA, IL-13, and TLR2 genes (94). In addition, the length and strength of the protective humoral immune response to the hepatitis B vaccination may also be impacted by sequence changes in genes related to pathogen identification, antigen processing and presentation, and cell differentiation and maturation. In this study, the frequencies of 53 known SNPs within 21 putative genes were compared between 24 non-responders and 46 responders. There was a significant (P < 0.05) correlation found between four SNPs in the IL-4, IL-4RA, IL-13, and TLR2 genes and the vaccination-induced serum anti-HBV response in the vaccinated individuals. When examined in conjunction with risk variables, including age and gender, two SNPs (rs1295686 within the 5-hydroxytryptamine receptor 2A (HTR2A) gene and rs1805015 within the solute carrier family 22 member 4 (SLC22A4) gene) also showed a significant connection with the vaccine-induced immune response (P < 0.05) by multivariable logistic regression analysis. Moreover, haplotype analysis showed that non-responders had a higher frequency of the AG haplotype, which is generated by SNPs rs1143633 (IL-1B; intron) and rs1143627 (IL-1B; intron), than did responders. Consequently, the status of the hepatitis B vaccine-induced protective humoral immune response was associated with SNPs in the cytokine/cytokine receptor genes and TLR2. Also, TLR2 rs3804100 polymorphisms may be protective factors for HBV-related HCC (94, 97). Genotyping assists in identifying HBV response opponents. The rs3804099 in TLR2 gene CT and TT genotypes have been demonstrated to influence hepatitis B progression (95). Xie et al. also conducted a haplotype study on the loci rs3804100 and rs3804099. They observed that the TT haplotype was importantly linked with lower HCC invasion. Mutually, the CC haplotype enhances the risk of HCC in patients over time (101). Using TLR2/MyD88/NF-κB signaling, STAT1-Ser727 phosphorylation, and IL-10-associate, partially autocrine mechanism, HBV increases monocyte production of inflammatory cytokines and inhibits IFN-α-stimulated stat1, stat2, and ch25h expression. In patients with CHB, coupling mRNA rates of IL-1 and IL-10 were usually more remarkable than in healthy people. To disrupt the antiviral innate immune response, HBV enhances the inflammatory response of monocytes while suppressing their IFN-α/β responsiveness. These efficacies have interceded through differential phosphorylation of Tyr701 and Ser727 of STAT1 (102). Researchers found that in a different study, rs4696480 and rs3804099 in TLR2 mutant carriers improved liver action parameters and reduced the rate of HBsAg, suggesting that TLR gene mutations may be able to lessen the inflammatory damage that results in long-term HBV diseases. Researchers showed that the mutations in rs3804099 and rs4696480 were related to an inhibited rate of TNF-α and IL-6 between patients with CHB compared to wild-type carriers. Additionally, the TLR2 mutant carriers had lower levels of pro-inflammatory cytokines and less inflammation due to the mutations in rs4696480 and rs3804099 (95).

### 5.2 TLR3

TLR3 is an innate immune system component that provides an early immunological response to foreign antigens by recognizing double-stranded RNA (dsRNA). Genetic variations, such as SNPs in TLR3, may contribute to an individual’s vulnerability to viral infections, such as HBV (103). Seven SNPs in the TLR3 gene are related to HBV, one of which is the rs1879026 polymorphism. The T allele of the rs1879026 polymorphism was shown to be substantially correlated with Saudis’ risk of contracting HBV infection in research conducted on a Saudi population (103). Nonetheless, a Chinese cohort revealed no correlation between the mutation and HBV-related liver illness. This shows that HBV susceptibility or ethnic variances may be influenced by the rs1879026 polymorphism, but not HBV-associated liver disorders (104). The haplotype GCGA of rs1879026, rs5743313, rs5743314, and rs5743315 were related to HBV in Saudi Arabians, as determined by haplotype analysis. The haplotype GT transporters of rs1879026-rs3775290 had a detracted risk of developing CHB, liver cirrhosis (LC), and HCC (41). However, a study conducted by Xu et al. did not find any significant link between the haplotype GT and HBV infection in a meta-analysis of diverse ethnic groups (41). There is disagreement over the relationship between the rs3775290 polymorphism and HBV (103). According to Goktas et al., patients with the TT genotype had higher HBV DNA levels than those with the CC and CT genotypes. This implies that a continued CHB condition may be associated with the TT genotype. A study conducted in Tunisian showed that the T allele became significantly related to chronic HBV infection and elevated the risk of HBV-associated HCC (105, 106). The TT genotype can inhibit CHB, HBV-associated LC, and HCC in Chinese populations but can decrease the likelihood of HBV transmission. The rs3775290 polymorphism on chromosome 4’s TLR3 gene can inhibit TLR3 activation, hindering pathogen recognition and weakening the immune response, thereby increasing the disease’s infectiousness (104). A meta-analysis validated the association between the rs3775291 polymorphism and HBV susceptibility, with the T allele and TT genotype enhancing the chances of HBV infection (107). Rong et al. found that individuals with the CT and TT genotypes had a 1.4-overlay and 2.3-fold greater probability of developing CHB, respectively, compared to those with the CC genotype. Similarly, they increased the CHB-associated high risk of acute chronic liver failure (ACLF) (108). A meta-analysis indicated that rs3775291 is a substantial risk factor for HBV-associated liver infection (107, 109). Li and Zheng discovered that it is associated with prolonged susceptibility to HBV-associated HCC (110). Chen et al. demonstrated, however, that polymorphism is likely an inhibitor factor for HBV-associated HCC. It was determined that the rs3775291 GG genotype was related to independence from HBsAg and HBeAg restriction. On the other hand, the A allele was linked to an increased risk of CHB (97, 111). The rs3775291 polymorphism was unrelated to HBV impotence in a Brazilian individual, possibly due to ethnic differences. This mutation causes the substitution of leucine with phenylalanine at position 412 of the TLR3 protein, and *in vitro* analysis has shown that it impairs the ability of TLR3 (112). TLR3 expression in HCC tissues may influence apoptosis and decrease HCC cell proliferation and angiogenesis (89).

### 5.3 TLR4

TLR4 initiates downstream signaling by using adaptor proteins TRIF, TRAM, and MyD88, which results in the activation of protein kinases and the production of IFN-β. TLR-mediated activation of the MyD88-dependent pathway regulates the production of genes necessary for adaptive immunity and inflammation, including pro-inflammatory IL-6 (113). rs1800769, rs2069845, and rs1880242 are SNPs in the TLR4 gene, which encodes protein. These SNPs have been linked to changes in the TLR4 gene, which produces the TLR4 protein. The innate immune system relies on this protein to recognize infections and trigger immunological responses (113). Polymorphism rs4986790 refers to a missense variant in the coding region that substitutes aspartic acid (69) for glycine (Gly) at the 299th position of the TLR4 protein (Gly). This molecule is found in TLR4’s extracellular domain near the myeloid differentiation protein-2 (MD-2) connection point (114, 115). In an investigation, there was a higher frequency of TLR3 rs3775290 CC genotype and TLR4 rs4986790 AA genotype among patients who had a stable response to the viral treatment, as compared to those who did not respond to the treatment. The study suggested that the TLR3 and TLR4 gene variants played a significant role in the treatment response, with TLR3 being more effective than TLR4 in reducing the viral load (106). In addition, rs1800796, rs2069845, and rs1880242 were associated with sustained viral response. Furthermore, researchers demonstrated that the common favorable IL-28B variant is essential for TLR-triggered antiviral preservation (113). It has been found that the AA wild-type genotype may have an impact on the severity of HBV-associated liver fibrosis in men, which can affect the early diagnosis of CHB (116). The TLR4 mRNA contains a polymorphism known as rs11536889 in its 3’-untranslated region (3`-UTR), which hinders LP-induced transmembrane signal transduction via post-transcriptional control of 3`-UTR. Moreover, this polymorphism is also responsible for preventing the recurrence of HBV following liver transplantation (117, 118). Based on a distinct study, it was observed that patients with CHB having CG and GG genotypes were independently associated with HCC. Further stratified studies revealed that the G allele of rs11536889 and the A allele of rs2149356 were identified as risk factors for cirrhosis in patients with CHB who do not consume alcohol (119, 120). A study by Abdelwahed et al. found that rs2069845 was associated with a reduced risk of CHB in a Saudi Arabian cohort (121).

### 5.4 TLR5

SNP rs5744174 is one of the TLR5 gene’s genetic mutations. It has been demonstrated that the SNP rs5744174 mutation affects how the body reacts to infections. Research has examined the relationship between this SNP and the severity or susceptibility to certain infectious illnesses (122). According to a study by Katrinli and colleagues, the rs5744174 polymorphism of the TLR5 gene in Turkish society is linked to CHB. The rs5744174 mutation converts Phe to Leu at position 616 of TLR5 (25). Due to differences in protein sequence and structure, this variation significantly impacts HBV sensitivity more than TLR5 expression levels (123). The TT genotype may protect against hepatitis B. Additionally, the T allele was observed to be associated with spontaneous conversion of blood HBeAg to TT, as well as higher IFN secretion levels (124, 125). Recent research reveals that genetic variations may lead to HBV-related severe liver damage (125). In addition, a study by Wu et al. found that the T allele at TLR5 rs5744174 was associated with earlier spontaneous HBeAg seroconversion in a Taiwanese cohort. This SNP may affect the IFN-γ production in response to HBV infection (126). In contrast, a study by Zhang et al. found no significant association between the rs5744174 SNP and CHB infection in a Chinese population. This SNP may not have a consistent effect on HBV infection across different ethnic groups (41).

### 5.5 TLR7

Through its ability to stimulate robust type I IFN production in pDCs, as well as to encourage B-cell activation and germinal center (94) responses, TLR7 is crucial for the induction of antiviral immunity. TLR7 is an X-linked gene-encoded single-stranded RNA receptor subject to strong purifying selection, indicating that it has a non-redundant biological role in host survival. TLR7 has been shown to play a crucial component in the GC responses to virus-like particles and ssRNA viruses (VLP). B-cell internal To counteract exogenous retrovirus, the GC responses depend on Myd88/TLR7 signaling (127). It has been demonstrated that TLRs, such as TLR7, promote B cell responses to immunogenic nucleic acids and immunological complexes (128) containing nucleic acids. Under normal circumstances, B cells can use nucleic acid recognition to react to early microbial assaults right away. Autoantibodies and autoimmunity, however, are encouraged when severe cellular or tissue damage takes place and B cell responses to an endogenous cellular nucleic acid are not inhibited. Many data points to the coordinated activation of TLR7 and the B cell receptor (BCR) in aberrantly activated B cells. By learning more about the possible molecular synergy between the TLR7 and BCR pathways in B cells, treatments that may be able to stop autoimmune states in patients can be developed (129). Zhu et al. conducted a study to determine the susceptibility and progression of CHB in Chinese individuals with altered TLR7 polymorphisms (rs179010, rs2074109, and rs179009). Another study revealed that Chinese men with CHB had a high frequency of the C allele of TLR7 rs179010, indicating an increased risk of CHB infection (23, 96). A study conducted by Al-Qahtani et al. found that the TLR7 rs179010 polymorphism is linked with a lower risk of CHB infection in Saudi Arabians (41). This polymorphism may impact the expression and function of TLR7, a receptor that detects viral RNA and triggers the immune response. The study suggests that the TLR7 rs179010 polymorphism may have an impact on the B-cell responses to HBV infection by altering the production of interferon-α (IFN-α) and immunoglobulin G (IgG) (47).

### 5.6 TLR8

While NK cells are the primary source of IFN-γ production, TLR8 agonists can selectively engage the immune system in the liver to increase the output of IFN-γ. It has been demonstrated that the TLR8 agonist GS-9688 may cause a long-lasting antiviral response in woodchucks infected with the hepatitis virus. GS-9688 is regarded as a possible medication to treat CHB since it may also cause the generation of cytokines in human immune cells to activate antiviral effector function. Many studies have demonstrated that HBV may block the TLR signaling pathway to evade the immune response, even if activating the TLR8 signaling pathway might cause NK cells to release IFN-γ (128). TLR8, a critical pyogenic bacteria sensor, is diminished by TLR signaling on the cell surface. Viral and bacterial infections are recognized by TLR8, an endosomal sensor of RNA breakdown products in human phagocytes (130). Uridine and small oligomers, both byproducts of RNA degradation, bind cooperatively at two separate locations in the N-terminal domain. The already-formed TLR8-dimer undergoes a conformational shift, allowing for the recruitment and signaling of MyD88 (131). Monocytes and myeloid dendritic cells are the primary cell types that express TLR8 (132–134). The researchers presented data obtained outside the living organism (ex vivo) that supports the theory that RNA from borrelia bacteria, delivered through endosomal vacuoles, activates TLR8. These investigations also showed that TLR8 is self-amplifying and solely responsible for inducing IFN-β in highly pure human monocytes through IRF-7, a similar signaling pathway linked to RNA viruses (135). The TLR8 SNP rs3764880 (Met1Val) modulates the translation of the two main TLR8 isoforms and plays an essential role in the immune reaction (136). In investigations, researchers screened about 250 Han Chinese individuals in Taiwan. They showed that a TLR8 Met/Val replacement at the start codon and a G>C alteration (rs3764880) at the TLR8−129 situation in the promoter zone (rs3764879) are in complete relation disequilibrium, with TLR8-129G/+1G allele frequencies of 84·0%. An SNP at the beginning codon (ATG>GTG, Met 1 Val) of TLR8 causes a frame-shift mutation, resulting in the organization of a truncated shape of TLR8 (1038 versus 1041 amino acids) comprising a three-amino-acid elimination at the N-terminus, which is as well as forecasted to be a part of the signal peptide (137, 138). However, the lack of animal models that accurately replicate the function of the TLR8 receptor is hindering research into its precise role in CHB. Furthermore, a thorough investigation of TLR8 expression patterns in CHB patients’ PBMCs is required, as is knowledge of how TLR8 is regulated in response to antiviral medication (42, 139, 140).

### 5.7 TLR9

Chihab et al. discovered three SNPs on the TLR9 gene related to HBV (93). The rs18708 G allele inhibits the development of HBV illnesses, whereas the AA genotype may significantly contribute to the progress of HBV infection relevant to progressive liver disease (AdLD). Moreover, it was substantially linked with HBV DNA load in genotype AA individuals with more DNA than the AG genotype. The rs573836 polymorphism was similarly linked to HBV DNA load, with the AA genotype having a lower DNA burden than the GG genotype (93). Additionally, Wu et al. demonstrated that the rs573836 CT genotype is linked with the initial spontaneous conversion of HBeAg (123). The rs5743836 polymorphism influences the transcription of TLR9 mRNA in the promoter region of the TLR9 gene. It has been discovered that its C allele increases promoter action, resulting in elevated TLR9 expression and thymosin 1, which signal and limit HBV growth via downstream cytokines (141). He et al. demonstrated that the rs352140 polymorphism was related to chronic HBV infection vulnerability and risk ratio for CT genotype carriers. It was insignificant to CC and TT genotypes (89). In an investigation, researchers examined the possibility of a relationship between an individual’s sensitivity to HBV intrauterine transmission and polymorphisms in TLR3 (rs3775290) and TLR9 (rs352140). The synonymous +2848 G > A polymorphism exists in exon two of the TLR9 gene and presumably influences expression at the mRNA rate. Following regulating risk agents, including maternal HBeAg, maternal HBV DNA, and mode of transfer, the minor allele ‘A’ of TLR9 was remarkably related to HBV intrauterine transmission capability, and the GA genotype preserved neonates from HBV intrauterine transmission. Carrier analysis showed that TLR9 GA+AA was mainly associated with intrauterine transmission capability (53, 142) (**Figure 3**).

## 6 Future perspective of TLRs impacting HBV pathogenesis

Previous studies have shown that intravenous injection of TLR4, TLR3, TLR7, TLR5, and TLR9 ligands/agonists lowers HBV replication in HBV transgenic mice (49). Furthermore, recent research has shown that HBV replication reduction in hepatoma cell lines and woodchuck models was facilitated by TLR2 activation (71). Synthetic chemicals like loxoribine and imidazoquinoline, as well as single-stranded viral RNAs, function as agonists for TLR7. TLR7 activates the MyD88-dependent pathway, which triggers transcription factors like JNK and NF-KB. This leads to the production of genes that secrete inflammatory cytokines and downstream targets. Researchers studied TLR7’s antiviral function during HBV infection and found it helps with viral clearance by manipulating host variables. They examined the cell cycle to see the effect of TLR7 activation on G1/S arrest caused by HBV. Researchers assessed the antiviral properties of imiquimod or R837, a synthetic compound that activates TLR7. R837 has been shown to have antiviral activity against various viruses, such as herpes simplex virus, human papillomavirus, and HBV (128). Furthermore, R848 uses TLR7 to activate pDCs, monocytes, and macrophages, which then produce a range of cytokines that facilitate both innate and acquired immunity (143) (**Table 1**). It was noted that when TLR7 was stimulated, HBV replication and the generation of viral proteins were suppressed. R837 likely activates the JNK pathway, which, therefore, causes the antiviral response. After R837 therapy, the repression mark on histone H3 (H3K9Me3) was shown to be downregulated in HBV-replicating HepG2.2.15 cells, the same number as healthy HepG2 cells. In addition, HBV causes a cell cycle stop in G1/S in HepG2.After being treated with the ligand, HepG2.2.15 cells were discharged. TLR7 has antiviral effects on HBV infection and shows promise as a therapeutic immunomodulator for treating this hepatotropic virus (144). Studies using TLR agonists as vaccine adjuvants show promise for various human vaccinations, including HBV vaccines. Vaccines with added TLR agonist(s) may activate particular TLR(s) to boost vaccination effectiveness without contributing directly to protective immunity. During the investigation of a therapeutic synthetic long peptide (SLP)-based vaccination for the treatment of CHB, TLR2-ligand conjugation of the prototype HBV-core SLP evoked functional patient T cell responses ex vivo, indicating that TLR agonists may also operate as possible adjuvants in HBV vaccines. PRR ligands have been shown in recent research to promote innate immunity that contributes to HBV control. Since there is evidence that suggests TLR agonists may be a helpful aid in the management of CHB, more study on their application in HBV therapies and vaccines is necessary (47). There has been conflicting evidence from research examining the significance of these polymorphisms in HBV infection. Therefore, their function in various populations and signaling pathways remains an area for further research. To decrease the infection rate and increase the treatment response, more effective procedures are required to overcome several challenges faced during HVB’s prevention, infection, and treatment. TLRs SNPs are yet to be fully understood about HBV infection. The mechanism of action of TLRs is not well understood since there has been so little research examining the effect of SNPs on HBV susceptibility. To fully comprehend the impact of TLR SNPs on HBV susceptibility and associated liver disorders, as well as to define its mode of action, functional investigations of SNPs in disease are required. Disease prevention, treatment, outcome, and prognosis monitoring will all benefit significantly from these findings. Because numerous genes commonly interact in the human body to impact illnesses, the clinical applicability of SNPs is currently restricted. For this reason, haplotype analysis, which incorporates data from several loci, should be prioritized in future research. We may be able to develop a model for predicting the likelihood of acquiring HBV infection with more studies. This will aid in the prevention, monitoring, and even treatment advice for HBV infection by increasing our capacity to anticipate the likelihood of HBV infection or the danger of advancement of associated liver disease via numerous TLR SNP (41, 53, 108, 117).

## 7 Conclusion

TLRs play a vital role in fighting HBV infection. Enhancing immune responses, mainly via the TLR signaling pathway, provides crucial protection against several pathogens, such as HBV, as they initiate subsequent inflammatory cascades and proinflammatory cytokines. In addition, research has demonstrated that alterations in the cellular immune response to HBV are connected with differential TLR2, TLR4, and TLR9 expression during the progression of HBV infections. Furthermore, TLR polymorphisms, genetic differences in signaling molecules implicated in TLR pathways, and downstream cytokines may influence HBV response. TLR SNPs can affect the body’s immune response to HBV infection and, therefore, the clinical outcome. For instance, the TLR2 -196 to -174 del SNP can increase the risk of chronic HBV infection and lead to a higher viral load. This is because it impairs the expression and signaling of TLR2. On the other hand, the TLR7 rs179008 SNP can decrease the risk of chronic HBV infection and lower the viral load. This is due to its ability to enhance the expression and signaling of TLR7. Similarly, the TLR9 rs352140 SNP can lead to spontaneous clearance of HBV infection and lower viral load, as it increases the expression and signaling of TLR9. The discovery of these gene expression profiles and SNPs may be significant for elucidating the pathogenic mechanisms of HBV susceptibility and the progression of related liver disease. By identifying these associated polymorphisms, we can efficiently advance the immune efficacy of vaccines. Simultaneously, other efficient prophylactic measures can be utilized for high-danger people with weak immune effectiveness to decrease the occurrence of viral infection efficiently. Additionally, genetic polymorphisms may assist in forecasting the clinical course of diseases.

## Author contributions

SS: Conceptualization, Data curation, Validation, Writing – original draft. AK: Writing – review and editing, Software. AY: Writing – review and editing, Software. HA: Project administration, Methodology, Writing – review and editing, Supervision.
